# Prevalence of school violence and use of alcohol and other drugs in adolescents^*^

**DOI:** 10.1590/1518-8345.2124.3110

**Published:** 2019-03-18

**Authors:** Maria Aparecida Beserra, Diene Monique Carlos, Maria Neto da Cruz Leitão, Maria das Graças Carvalho Ferriani

**Affiliations:** 1 Universidade de Pernambuco, Faculdade de Enfermagem Nossa Senhora das Graças, Recife, PE, Brazil.; 2 Universidade de São Paulo, Escola de Enfermagem de Ribeirão Preto, PAHO/WHO Collaborating Centre for Nursing Research Development, Ribeirão Preto, SP, Brazil.; 3 Escola Superior de Enfermagem de Coimbra, Coimbra, Portugal.

**Keywords:** Adolescent Behavior, School Health, Violence, Street Drugs, Underage Drinking, Smoking, Comportamento do Adolescente, Saúde Escolar, Violência, Drogas Ilícitas, Consumo de Álcool por Menores, Hábito de Fumar, Conducta del Adolescente, Salud Escolar, Violencia, Drogas Ilícitas, Consumo de Alcohol en Menores, Hábito de Fumar

## Abstract

**Objective:**

to analyze the school violence suffered and practiced and its association with the use of alcohol and other drugs in adolescents between 12 and 18 years old.

**Method:**

the study sample consisted of 643 adolescents enrolled in six schools, who answered two self-administered questionnaires: “Global School-based Student Health Survey” and “Violence in School”. Statistical analysis was performed using the chi-square test and the degree of association between the variables was analyzed using the prevalence ratio.

**Results:**

the prevalence of school violence suffered and practiced was 62.2% and 51.9%, respectively. About 44.6% of the aggressors said they did not want to change their behavior. There was an expressive prevalence of alcohol use (16.5%), tobacco (15.7%) and illicit drugs (6.8%), and drunkenness (12.6%). There was a significant association between the violence suffered and the age group of 12 to 14 years old (p=0.001); (p=0.011) and education level in elementary school (p<0.001). In mothers with less than eight years of studies, the association was significant for the violence practiced (p=0.002).

**Conclusion:**

the study contributes to the aspects involved in school violence, which can subsidize actions and policies in this area.

## RESUMO

**Objetivo:**

analisar a violência escolar sofrida e praticada e a sua associação com o uso de álcool e outras drogas entre adolescentes com 12 a 18 anos de idade.

**Método:**

a amostra do estudo foi composta por 643 adolescentes matriculados em seis escolas, que responderam a dois questionários autoaplicáveis: “*Global School-based Student Health Survey*” e “Violência na Escola”. A análise estatística foi realizada utilizando-se o teste Qui-quadrado e o grau de associação entre as variáveis analisado por meio da razão de prevalência.

**Resultados:**

as prevalências de violência escolar sofrida e praticada foram de 62,2% e 51,9%, respectivamente. Cerca de 44,6% dos agressores afirmaram não querer mudar seu comportamento. Houve prevalência expressiva do uso de álcool (16,5%), tabaco (15,7%), drogas ilícitas (6,8%) e de embriaguez (12,6%). Houve associação significativa entre a violência sofrida e a faixa etária de 12 a 14 anos (p=0,001), sexo masculino (p=0,011) e grau de escolaridade em ensino fundamental (p<0,001). Em mães com menos de oito anos de estudo, a associação foi significativa para a violência praticada (p=0,002).

**Conclusão:**

o estudo traz contribuições para os aspectos envolvidos na violência escolar, que podem subsidiar ações e políticas nesse âmbito.

## RESUMEN

**Objetivo:**

analizar la violencia escolar sufrida y practicada y su asociación con el uso de alcohol y otras drogas entre adolescentes de 12 a 18 años de edad.

**Método:**

la muestra del estudio fue compuesta por 643 adolescentes matriculados en seis escuelas, que respondieron a dos cuestionarios autoaplicables: *“Global School-based Student Health Survey*” y “Violencia en la Escuela”. El análisis estadístico fue realizado utilizando el test Chi-cuadrado y el grado de asociación entre las variables fue analizado por medio de la razón de prevalencia.

**Resultados:**

las prevalencias de violencia escolar sufrida y practicada fueron de 62,2% y 51,9% respectivamente. Alrededor del 44,6% de los agresores afirmaron no querer cambiar su comportamiento. Se observó una prevalencia expresiva del uso de alcohol (16,5%), tabaco (15,7%) y drogas ilícitas (6,8%), y de embriaguez (12,6%). Se observó una asociación significativa entre la violencia sufrida y el grupo de edad de 12 a 14 años (p=0,001); sexo masculino (p=0,011) y grado de escolaridad en enseñanza primaria (p<0,001). En madres con menos de ocho años de estudio, la asociación fue significativa para la violencia practicada (p=0,002).

**Conclusión:**

el estudio aporta contribuciones a los aspectos envueltos en la violencia escolar, que pueden subsidiar acciones y políticas en este ámbito.

## Introduction

Violence is a problem that infiltrates society, threatening the healthy development of people. For these issues, it needs to be studied transversally. It is responsible for a large proportion of deaths in several countries, particularly among children, adolescents, and young adults. The exposure to violence can cause immediate physical injury that health professionals can handle. However, it can result in physical and mental health problems that are not often apparent to these professionals. Also, violence directly affects expenditures on health care; and indirectly, it generates stagnation of economic development, and increases inequities and deteriorates human capital^1^.

Violence is materialized in several social spaces and, in recent years, it has been frequent in the school environment. This contradicts the role of the school. School violence is expressed in a more explicit perspective of violence, such as aggression between individuals, and in the symbolic violence that occurs through the rules, norms and cultural habits of an unequal society^2^.

Adolescent development involves a progressive independence and autonomy of the family, a greater association with the pairs, the formation of the identity, and the physiological and cognitive maturation. This whirlwind of changes allow the adolescent to open new horizons and experience new behaviors, some of which involve present and future health risks, such as the use of psychoactive substances such as alcohol and illicit drugs^3^ ^-^ ^4^. The family emerges as essential in this debate by its implication in these behaviors presented by the adolescents, besides the consideration of the ecological gaze for the understanding of the violence^5^ ^-^ ^7^. Recent literature review has brought the factors associated with the perpetuation of violence, with poor impulse control; use of alcohol or other drugs; unhealthy relationships between caregivers and children; family economic stress; exposure to community violence; increase in social inequities were factors strongly correlated with the occurrence of violence among adolescents and young people^1^.

The present study lists as risk behaviors factors related to adolescents’ lifestyle, such as alcohol and drug use, as well as to violent behaviors considered as predictors of the violent act. In Brazil, the analysis of these variables by the National Survey of School Health (PeNSE) conducted in 2009, using data from the Brazilian Institute of Geography and Statistics^8^, revealed that 71.4% of schoolchildren have ever tried alcoholic beverages. The use of alcohol in adolescence is a factor of exposure to health problems in adulthood, as well as significantly increasing the risk of the individual becoming an abusive consumer throughout life^9^.

According to data from PeNSE conducted in 2012, 15.9% of students suffered physical aggression in the last twelve months before conducting the research; in the PeNSE of 2015, there was an increase in the percentage of aggression to 17.3%^3^ ^-^ ^4^ ^,^ ^10^. Comparing the PeNSE data for 2009, 2012 and 2015, there is an increase in prevalence in almost all variables related to situations of violence involving students, as well as the severity of violence experienced by adolescents^11^. On the other hand, there is an association between the adoption of a healthy lifestyle by young people and the decrease in the relative risk of being frequently intimidated and victimized^12^.

In this dimension, it is fundamental to work the behavior of adolescents in the school environment; aiming to identify the behavioral risk factors and protect these individuals are exposed. Therefore, the present study aims to identify the prevalence of violence suffered and practiced by adolescents in the school context and to analyze its association with the use of alcohol, tobacco, and other drugs. The proposed approach is shown as an emerging health issue, considering the importance that the school institution occupies in adolescents’ lives, as well as the seriousness of the violent incidents that occur in this environment involving these actors. It is emphasized that adolescence constitutes a “key period” of human development, in which actions that promote a healthy adult life can be implemented^13^.

## Methods

This study is part of a study carried out with the objective of studying the violence suffered and/or practiced by adolescents in the school context and the associated individual and environmental factors, in public schools in the city of Recife, state of Pernambuco, Brazil. The study was approved by the Research Ethics Committee of the University of Pernambuco, on March 20, 2012, and the study protocol was registered under CEP/UPE 10/12. Participating subjects spontaneously consented to participate in the study, which was confirmed by the signing of the Term of Assent by them and Free and Informed Consent Form by their parents or legal guardians. The Regional Education Management of Recife Norte, of the Education Department of Pernambuco, also granted the authorization to carry out this study in the State Schools. The research project was presented previously at the parents’ meeting marked by the school board, in which the objectives of the study were exposed by the first author.

This is a cross-sectional study with an estimated population of 4,905 students enrolled in six state schools in a district of the municipality studied. The sample consisted of 643 adolescents, aged between 12 and 18 years old, enrolled in Elementary and Middle School, in 2013, and who accepted to participate in the research. Adolescents who were separated on medical leave, maternity leave or suspended; and those who did not meet the inclusion criteria were excluded. The probabilistic sampling method stratified by school was used to determine the sample size and to select the individuals, considering the estimated population size of 4,905 students enrolled in the six state schools of the studied neighborhood; the margin of error of 5.0%; the 95% confidence that the margin of error is not exceeded; the expected proportion of 50.0% for each response category, which maximizes sample size. The total number of students in the sample (643) was distributed proportionally between the six selected schools and the three school shifts. All calculations for sample determination were performed through the Epi-Info program version 6.04d for DOS.

The data were collected from 02/07/2013 to 06/05/2013 by a nurse and author of this article, using two self-administered questionnaires already validated in Brazil. The first questionnaire, containing sociodemographic and behavioral questions, aims to evaluate the exposure to health risk behaviors in adolescents, being denominated Global School-Based Student Health Survey^14^ ^-^ ^15^. The second questionnaire has school violence as its central theme and its objective is to evaluate the violence suffered, practiced and/or witnessed by adolescents within the school context^16^ ^-^ ^17^. The data collected were scanned in spreadsheets with double entry and the data bank was validated by a third person.

The variable outcome of the study was the occurrence of violence suffered and/or practiced in the school context, in the last two weeks that preceded the study. The type of violence was explored, who was the aggressor, who was the victim, the place where the violence occurred, how many times they were an aggressor or victim, whether they continue to attack or be attacked. The explanatory variables were selected from the Global School-Based Student Health Survey^14^ ^-^ ^15^, being age, gender, education level, residing with the mother, residing with the father, length of study of the mother, alcohol consumption and other illicit drugs (age at the beginning of consumption, number of doses of alcohol, drunkenness, where the drug was obtained).

The results were analyzed through absolute and percentage distributions. To evaluate the existence of an association between two categorical variables, the Chi-square statistical test was used and when the conditions for its use were not met, the Fisher’s exact test was used. To evaluate the degree of association between the variables, the prevalence ratio (PR) and the respective confidence interval (CI) were obtained. The margin of error was 5%, and the degree of association between the variables was assessed by the prevalence ratio and the respective confidence interval (CI 95.0%). The Statistical Package for the Social Sciences (SPSS), version 21, was used to carry out all the statistical analyzes of the results.

## Results

The main sociodemographic characteristics of the sample analyzed were: slightly more than half (56.5%) of the adolescents were between 15 and 18 years old - the others (43.5%) were between 12 and 14 years old; most of them were female (64.2%), single (93.6%), did not work (87.9%), and were self-considered as non-white (82.3%), with predominance of brown/mulatto/brunette (70.6%). Regarding the education level, just over half of the adolescents (54.1%) attended high school; just over half (52.3%) of the mothers of those surveyed had 8 to 11 years of study. As to housing, almost all (95.5%) of the adolescents lived in the urban region, with their mother (89.7%), and just over half (56.8%) lived with their father.

The analysis of the prevalence of violence in the school revealed that most (62.2%) of the adolescents were victim of aggression by their classmates or others in the school environment in the two weeks before the survey (Table 1). The most frequent type of aggression was verbal (54.2%), and most of the adolescents were beaten once (79.8%) by one person (65.5%); however, about 12.5% of adolescents continued to be assaulted by the same people. Almost all of the aggressors were classmates (96.0%), and the classroom was the school site where the most cited acts of violence occurred (45.9%). Nearly half (51.3%) of the cases of aggression were witnessed by third parties, who, for the most part, did not take any action (36.7%) or supported the adolescent victim of violence and advised him to move away of the aggressor (27.1%).

**Table 1:** Absolute and relative distribution of the study population according to the issues related to the fact that they were victims of violence in the school*. Recife, PE, Brasil, 2013

**Variable**	**n**	**%**
**Have you ever felt victimized by peers or other people at school?** ^**†**^		
**Yes**	**400**	**62.2**
**No**	**243**	**37.8**
**Types of aggression suffered** ^**‡**^		
**Physics**	**129**	**24.0**
**Verbal**	**292**	**54.2**
**Psychological**	**44**	**8.2**
**Patrimonial**	**26**	**4.8**
**Sexual**	**26**	**4.8**
**Other aggressions**	**22**	**4.1**
**How many times have you been beaten or persecuted?** ^**§**^		
**Once**	**319**	**79.8**
**Twice or more times**	**81**	**20.2**
**In which school did these situations occur?** ^**||**^		
**Classroom**	**224**	**45.9**
**Courtyard**	**83**	**17.0**
**Corridors and/or stairs**	**88**	**18.0**
**Dining hall**	**26**	**5.3**
**Physical Education Space**	**11**	**2.2**
**Bathroom**	**18**	**3.7**
**Other**	**38**	**7.8**
**In these situations, how many people have you been beaten?** ^**§**^		
**One**	**262**	**65.5**
**Two**	**94**	**23.5**
**Three or more**	**44**	**11.0**
**In these situations, what people have you been assaulted?** ^**¶**^		
**Classmates**	**384**	**96.2**
**School workers**	**19**	**4.8**
**Are you still being beaten/persecuted by these people?** ^**§**^		
**Yes**	**50**	**12.5**
**No**	**350**	**87.5**
**Has anyone witnessed such situations?** ^**§**^		
**Yes**	**205**	**51.3**
**No**	**195**	**48.8**
What did the people who witnessed these situations do?**		
**Nothing**	**88**	**36.7**
**Escape /was afraid**	**8**	**3.3**
**Looked for an adult**	**19**	**7.9**
**Asked the aggressor to stop**	**25**	**10.4**
**Supported the aggressor/ Smiled at the situation**	**30**	**12.5**
**Supported you / Advised you to move away from the aggressor**	**65**	**27.1**
**Approached to see / Another attitude**	**5**	**2.1**

*In the two weeks before the survey; †Percentage calculated in the total number of answers, n=643; ‡Considering that the same participant cited more than one answer option, the basis for calculating the percentages and not the total answers was recorded, where n=539; §Percentage calculated in the total answers, n= 400; ||, ¶, **Considering that the same participant cited more than one answer option, the basis for calculating the percentages and not the total answers was recorded, being || n= 488, ¶ n= 403 and ** n= 240

When questioned about having committed acts of violence in the school environment, slightly more than half (51.9%) of adolescents said they had aggressive attitudes towards a student, teacher or any other school worker. This subgroup of aggressors of violence practiced verbal aggression (55.6%) and physical aggression (33.5%), generally alone (65.0%), that is, without the help of other individuals. The main place of occurrence was within the classroom (49.7%), followed by the courtyard (16.8%) and corridors and/or stairs (16.8%), against a roommate (94.37%). Most adolescents had aggressive attitudes only once (77.8%), and most of them had twice (10.5%) or three or more times (11.7%), with 6.9% of adolescents having assaulted and/or persecuted the same person more than once.

The main reasons that lead to the violent act were joking (44.8%), irritation (15.7%) and revenge (15.2%). Similar to the previously report, about half (53.6%) of the cases of aggression were witnessed by third parties, who, for the most part, did not take any action (47.4%) or support the aggressor and/or smiled at the situation (26.3%) Just over half of the aggressors (55.4%) stated that they wanted to behave differently towards those who were attacked, not responding to provocations (25.6%) or controlling themselves (24.7%). However, 28.4% of the aggressors said they did not want to change their behavior.

The analysis of the prevalence of alcohol and other drug use (Table 2) showed that 16.5% of the adolescents consumed at least one dose of alcohol-containing beverage within the thirty days before the study. In this subgroup, there was predominance of adolescents who had consumed 2 doses (23.6%) or 4 or more (31.1%) doses of alcoholic beverage. About half (49.1%) of the adolescents acquired the drink consumed in a bar/restaurant/supermarket/street vendor, and about a third (35.8%) got it with friends. When asked if they had been drunk during their lifetime, most (82.4%) of the adolescents answered negatively to the question, while 12.6% had already been drunk one to two times. Almost all (93.2%) of the adolescents never consumed drugs like *loló*, glue, perfume, marijuana, crack and cocaine; and most who answered affirmatively, 5.3% used drugs of this type one to two times.

**Table 2:** Absolute and relative distribution of the study population according to the issues related to the consumption of alcohol and other illicit drugs. Recife, PE, Brasil, 2013

**Variable**	**n**	**%**
In the last 30 days, have you consumed at least one drink containing alcohol?* ^**,†**^		
**Yes**	**106**	**16.5**
**No**	**537**	**83.5**
**How many doses did you drink per day?** ^**†,‡**^		
**Less than 1 dose**	**13**	**12.3**
**One dose**	**19**	**17.9**
**Two doses**	**25**	**23.6**
**Three doses**	**16**	**15.1**
**4 doses or more**	**33**	**31.1**
**How did you get the drink you consumed?** ^**†,‡**^		
**Bought it in a bar/Restaurant/Supermarket/Street vendor**	**52**	**49.1**
**Got it with your friends**	**38**	**35.8**
**Got it in their house**	**11**	**10.4**
**Stole it/Got it in some other way**	**5**	**4.7**
During your life, how many times have you been drunk?*		
**None**	**530**	**82.4**
**1 to 2**	**81**	**12.6**
**3 or more**	**32**	**5.0**
During your life, how many times have you used illicit drugs?*		
**None**	**599**	**93.2**
**1 to 2**	**34**	**5.3**
**3 or more**	**10**	**1.6**

*Percentages calculated in the total answers (n= 643); †The questions are about the period of 30 days before the research; ‡Percentages calculated in the total of affirmative answers to the first question (n= 106)

The bivariate analysis of the association between the variables answer (violence suffered and violence practiced) and sociodemographic variables (Table 3 and 4) showed a significant association between the violence suffered and the age group of 12 to 14 years old (p=0.001, RP=1.35); (p=0.011, PR=1.17) and education level in elementary school (p<0.001, PR=1.33). The practice of violence decreased due to the increase in the mother’s education; in mothers with less than eight years of study, the association was significant (p=0.002, PR=1.42).

**Table 3:** Bivariate analysis and crude prevalence ratios of the index of violence suffered, according to sociodemographic data. Recife, PE, Brasil, 2013

	**Violence suffered**						
**Variable**	**Yes**			**No**		p-value*	**PR** ^**†**^ **(CI** ^**‡**^ **a 95%)**
	**n**	**%**		**n**	**%**		
**Age group**							
**12 to 14**	**204**	**72.9**		**76**	**27.1**	**< 0.001** ^**§**^	**1.35 (1.20 a 1.52)**
**15 to 18**	**196**	**54.0**		**167**	**46.0**		**1.00**
**Gender**							
**Male**	**158**	**68.7**		**72**	**31.3**	**0.011** ^**§**^	**1.17 (1.04 a 1.32)**
**Female**	**242**	**58.6**		**171**	**41.4**		**1.00**
**Marital status**							
**Single**	**373**	**62.0**		**229**	**38.0**	**0.610**	**1.00**
**Married/Stable union**	**27**	**65.9**		**14**	**34.1**		**1.06 (0.85 a 1.34)**
**Education level**							
**Elementary school**	**212**	**71.9**		**83**	**28.1**	**0.001** ^**§**^	**1.33 (1.18 a 1.50)**
**High school**	**188**	**54.0**		**160**	**46.0**		**1.00**
**Residing with**							
**Parents**	**210**	**63.3**		**122**	**36.7**	**0.817**	**1.10 (0.81 a 1.49)**
**Only with the mother**	**149**	**60.8**		**96**	**39.2**		**1.06 (0.78 a 1.44)**
**Only with the father**	**22**	**66.7**		**11**	**33.3**		**1.16 (0.79 a 1.69)**
**Other person**	**19**	**57.6**		**14**	**42.4**		**1.00**
**Education level of the mother**							
**Less than 8 years**	**124**	**66.7**		**62**	**33.3**	**0.324**	**1.09 (0.92 a 1.30)**
**8 to 11 years**	**202**	**60.1**		**134**	**39.9**		**0.98 (0.83 a 1.16)**
**12 or more**	**74**	**61.2**		**47**	**38.8**		**1.00**

*P-value determined by Pearson’s Chi-square test; †PR - Prevalence ratio; ‡CI - Confidence Interval of 95%; §Significant difference of 5.0%

**Table 4:** Bivariate analysis and crude prevalence ratios of the violence rate practiced, according to sociodemographic data. Recife, PE, Brasil, 2013

	**Violence practiced**						
**Variable**	**Yes**			**No**		p-value*	**PP** ^**†**^ **(IC** ^**‡**^ **to 95%)**
	**n**	**%**		**n**	**%**		
**Age group**							
**12 to 14**	**163**	**58.2**		**117**	**41.8**	**0.005** ^**§**^	**1.24 (1.07 a 1.43)**
**15 to 18**	**171**	**47.1**		**192**	**52.9**		**1.00**
**Gender**							
**Male**	**143**	**62.2**		**87**	**37.8**	**< 0.001** ^**§**^	**1.34 (1.16 a 1.55)**
**Female**	**191**	**46.2**		**222**	**53.8**		**1.00**
**Education level**							
**Elementary school**	**177**	**60.0**		**118**	**40.0**	**< 0.001** ^**§**^	**1.33 (1.15 a 1.54)**
**High school**	**157**	**45.1**		**191**	**54.9**		**1.00**
**Working**							
**Yes**	**39**	**50.0**		**39**	**50.0**	**0.714**	**1.00**
**No**	**295**	**52.2**		**270**	**47.8**		**1.04 (0.83 a 1.32)**
**Residing with**							
**Parents**	**174**	**52.4**		**158**	**47.6**	**0.841**	**1.00**
**Only the mother**	**123**	**50.2**		**122**	**49.8**		**0.96 (0.82 a 1.13)**
**Only the father**	**19**	**57.6**		**14**	**42.4**		**1.10 (0.81 a 1.50)**
**Other person**	**18**	**54.5**		**15**	**45.5**		**1.04 (0.75 a 1.44)**
**Education level of the mother**							
**Less than 8 years**	**116**	**62.4**		**70**	**37.6**	**0.002** ^**§**^	**1.42 (1.13 a 1.79)**
**8 to 11 years**	**165**	**49.1**		**171**	**50.9**		**1.12 (0.89 a 1.41)**
**12 or more**	**53**	**43.8**		**68**	**56.2**		**1.00**

*P-value determined by Pearson’s Chi-square test; †PR - Prevalence ratio; ‡CI - Confidence Interval of 95%; §Significant difference of 5.0%

The bivariate analysis of the association between the answer variables (alcohol, tobacco and illicit drug use) and sociodemographic variables (age and gender of adolescents, violence suffered and practiced) (Table 5) showed the existence of a significant association between the age group of adolescents with the variables: alcohol use, tobacco use and illicit drug use. It is possible to emphasize that the percentage of adolescents who practiced the acts related to these variables were about three times higher in the age group of 15 to 18 years old than in the age group of 12 to 14 years old. In this last age group, non-use of alcohol was significantly associated (p<0.001, PR=1.00), as the non-use of tobacco (p<0.001, PR=1.00) and illicit drugs (p=0.014, PR=1.00). There was no significant association between the cited answer variables and adolescents’ gender, violence suffered and violence practiced, despite the prevalence of women in alcohol, tobacco, and illicit drug use.

**Table 5:** Bivariate analysis and crude prevalence ratios of alcohol, tobacco, and illicit drug use, according to age, gender, and violence suffered and violence practiced. Recife, PE, Brasil, 2013

**Variable**	**Yes**		**No**		p-value*	**PR** ^**†**^ **(CI** ^**‡**^ **to 95%)**
	**n**	**%**	**n**	**%**		
**Use alcohol**						
**Age group**						
**12 to 14**	**17**	**8.3**	**187**	**91.7**	**< 0.001** ^**§**^	**1.00**
**15 to 18**	**54**	**27.6**	**142**	**72.4**		**3.31 (1.99 a 5.50)**
**Gender**	** **					
**Male**	**28**	**17.7**	**130**	**82.3**	**0.990**	**1.00**
**Female**	**43**	**17.8**	**199**	**82.2**		**1.00 (0.65 a 1.54)**
**Violence suffered**						
**No**	**71**	**17.8**	**329**	**82.2**	**0.267**	**1.23 (0.85 a 1.79)**
**Yes**	**35**	**14.4**	**208**	**85.6**		**1.00**
**Violence practiced**						
**No**	**58**	**17.4**	**276**	**82.6**	**0.532**	**1.12 (0.79 a 1.59)**
**Yes**	**48**	**15.5**	**261**	**84.5**		**1.00**
**Use of tobacco**						
**Age group**						
**12 to 14**	**15**	**7.4**	**189**	**92.6**	**< 0.001** ^**§**^	**1.00**
**15 to 18**	**51**	**26.0**	**145**	**74.0**		**3.54 (2.06 a 6.08)**
**Gender**						
**Male**	**25**	**15.8**	**133**	**84.2**	**0.768**	**1.00**
**Female**	**41**	**16.9**	**201**	**83.1**		**1.07 (0.68 a 1.69)**
**Violence suffered**						
**No**	**66**	**16.5**	**334**	**83.5**	**0.479**	**1.15 (0.79 a 1.67)**
**Yes**	**35**	**14.4**	**208**	**85.6**		**1.00**
**Violence practiced**						
**No**	**56**	**16.8**	**278**	**83.2**	**0.443**	**1.15 (0.80 a 1.65)**
**Yes**	**45**	**14.6**	**264**	**85.4**		**1.00**
**Use of illicit drugs**						
**Age group**						
**12 to 14**	**8**	**3.9**	**196**	**96.1**	**0.014** ^**§**^	**1.00**
**15 to 18**	**20**	**10.2**	**176**	**89.8**		**2.60 (1.17 a 5.77)**
**Gender**						
**Male**	**11**	**7.0**	**147**	**93.0**	**0.981**	**1.00**
**Female**	**17**	**7.0**	**225**	**93.0**		**1.01 (0.49 a 2.10)**
**Violence suffered**						
**No**	**28**	**7.0**	**372**	**93.0**	**0.840**	**1.06 (0.59 a 1.92)**
**Yes**	**16**	**6.6**	**227**	**93.4**		**1.00**
**Violence practiced**						
**No**	**25**	**7.5**	**309**	**92.5**	**0.503**	**1.22 (0.68 a 2.17)**
**Yes**	**19**	**6.1**	**290**	**93.9**		**1.00**

*P-value determined by Pearson’s Chi-square test; †PR - Prevalence ratio; ‡CI - Confidence Interval of 95%; §Significant difference of 5.0%

## Discussion

The prevalence of violence in the school context, in the condition in which the adolescents were victims of the aggression, was 62.2%. In Brazil, the estimated prevalence is 10% to 70% and, in the international scenario, from 8% to 60%^18^ ^-^ ^19^. In spite of the diversification of the prevalence of violence in the school context, these studies highlight common central issues: school space is not immune to the presence of violence, and adolescents’ exposure to school violence is a worldwide concern that has led researchers from several areas of knowledge to investigate the occurrence of this phenomenon, such as health and education. A recent study addressing this theme identified the fragility or absence of institutional links to address school violence, given the need to think about this problem in an expanded and articulated network^20^.

The main protagonists of the violence were the classmates (96.2%). In general, episodes of violence in the school involving only students are those that occur more frequently and have greater visibility for the different actors in this context^21^. Violent acts occurred predominantly in the classroom (45.9%), corroborating with other studies^19^ ^-^ ^21^. This space that should be protected and have a mediating figure is pointed out as a locus of violence. The literature is still incipient in the discussion of this issue, pointing out the need for a directed view at this specificity. Adolescents are incorporated into multiple contexts and each of these contexts interacts with individual characteristics in a way that exacerbates or attenuates the association between these individual characteristics and aggression or victimization. The main source of peer group formation is in the physical setting in which most children are centered^22^.

Recent research indicates that 70-80% of victims of violence and their bullies are in the same class at school^23^. Also, school violence is still associated with males, corroborating studies in the area about bullying^24^ ^-^ ^25^. However, the literature has shown that the girls have attacked as much as the boys, and in a direct way^17^. Students enter the school setting with a combination of internal and interpersonal strengths and weaknesses that influence their academic success and behavioral functioning. Prevention and intervention efforts should take a multi-component approach to effectively addressing behavioral concerns. Programs focused on rephrasing normative beliefs while using skill-building techniques, may be best suited for improving behavior in the classroom and throughout the school^26^.

Concerning the violence among adolescents in the school context, two worrying facts revealed by the present study were the high incidence (44.6%) of aggressive adolescents who stated that they did not want to change their violent behaviors and the significant percentage (26.3%) of adolescents who, having witnessed the aggression, supported the aggressor and laughed at the situation. Such attitudes of students who witness violence may be motivated by the desire to improve their peer status, as aggressors become popular, or out of fear, in an attitude of self-protection. The literature has pointed out the importance of looking at the spectators of violence among adolescents, who have been neglected by research and interventions; a climate of peace culture can act as a significant protective factor for violence among adolescents^27^ ^-^ ^29^.

The family context emerges as preponderant in the discussion of school violence; the family is shown as the first environment of socialization and internalization of emotions and behaviors, which will be experienced in other spaces^6^ ^-^ ^7^. Although the instruments used in this study did not allow determining the number of nuclear families among the adolescents, it was identified that most of the adolescents live with the mother. This may indicate single-parent families and/or the existence of family conflicts. Literature review has brought about that family characteristics are determinant for the involvement of adolescents with bullying, especially single parenting; this fact is related to a possibility of less time for parent-child interaction and greater family stress^5^.

Another aspect related to family relationships was the significant association between violence practiced and low level of education of the mother; this issue corroborates literature in the area, “pointing out that education enhances a culture of tolerance and respect for human rights”^30^. In a study about the meanings attributed to violence in the school context by teachers, they emphasized the importance of work and a directed look at the family, given the relevance of this institution to the formation and behavior of children and adolescents in other social environments^31^. Despite this importance, the literature corroborates that family-school integration is still a challenge^20^ ^,^ ^31^.

The violence suffered by the adolescents showed association between 12 and 14 years old. The literature corroborates such findings regarding the various types of violence to which adolescents are exposed and warns that these adolescents may live and mean violence in different ways^27^ ^,^ ^32^. Therefore, it suggests the development of instruments that identify and allow more targeted interventions for these age groups.

Regarding health risk behaviors in the present study, 5.3% of adolescents used drugs and 16.5% consumed alcoholic beverages; in this last group, 12.6% of the individuals already got drunk. The PeNSE of 2012 revealed that 26.1% of 9^th^ grade students consumed alcoholic beverages in the last 30 days before the survey, with drunkenness reported by 21.8% of respondents^33^. The PeNSE of 2015 showed that 55.5% of the students in the 9^th^ grade of elementary education responded positively, experimenting with alcoholic beverages^10^. Comparing the prevalence of alcohol consumption reported in different studies, the values often differ according to the age range of the subjects.

It is important to emphasize that youth cultures are articulated with the construction and adoption of styles. Based on this understanding, alcoholic beverage has assumed a symbolic place for adolescents, and it is possible that the threshold of tolerance may be constituting a dispute, also symbolic, where those who have more tolerance to drink may be at an advantage over others, considered as more fragile. However, the greater tolerance concerns the need for progressively larger amounts of substance to produce the desired effect, increasing the risk of intoxication^34^. Also, this aspect hurts the public policies, denouncing the sale of alcoholic beverages to adolescents under 18 years old. This fact is forbidden in Brazil. While it is illegal to sell alcoholic beverages to young people under 21, the alcohol and tobacco industries create an environment in which the consumption of these dangerous products is acceptable and, within some groups of adolescents, is expected. Many health promotion efforts to reduce health risks emphasize individual behavior change and ignore the critical role of environmental and social factors. Studies suggest that efforts to reduce alcohol consumption by young people should incorporate population-based policies for coping with excessive alcohol consumption in adults as part of a comprehensive approach to preventing alcohol-related harm^35^.

Another health risk behavior analyzed in this study was tobacco consumption. About 15% of adolescents already smoked some form of tobacco, and 61.4% of them did it for the first time when they were between 10 and 15 years old. The results of PeNSE conducted in 2015 showed that the prevalence of cigarette experimentation in adolescents in the 9^th^ grade of elementary school in public and private schools in Brazil was 18.49%, with the highest frequency of experimentation observed in the Southern Region (24.9%) and the lowest in the Northeast Region (14.2%) in Brazil^10^.

Tobacco is one of the most important determinants for triggering chronic diseases. According to the World Health Organization, tobacco leads the ranking of causes of preventable deaths in the world. Also, the early onset of smoking is associated with an increased chance of using other substances harmful to health, such as alcohol and illicit drugs^36^. In the city of Belo Horizonte, Minas Gerais, Brazil, the prevalence of smoking among adolescents and young adults was 11.7%, and the main factor associated with tobacco use was excessive alcohol consumption^37^.

The World Health Organization also highlights that the use of alcohol and tobacco by individuals under 14 is associated with increased risk of school dropout, aggression, suicide and alcohol intoxication, and the mental health, with a number of negative consequences in the short and long-term^36^. Systematic review of literature on the influence of the social network on the smoking behavior of adolescents has brought about that social isolation is related to smoking among adolescents. Also, peer selection and influence contribute to the initiation and maintenance of smoking in adolescents^38^. Thus, the importance of actions among peers is observed, essentially in the privileged school context. In addition, the look at family support is relevant. A study that analyzed predictors of smoking in young adults from smokers and non-smokers showed that low perceived levels of family social support were a critical factor for smoking among smokers and non-smokers. The association between alcohol use and depressive symptoms was also relevant^39^.

Finally, some limitations of the study are highlighted. Despite the reliability and validity of the research, the transversal design does not allow specific temporal analyzes for school violence. The study site was a specific neighborhood, which preserves particularities, limiting the generalization of the findings. The instruments used also do not allow bringing some familiar characteristics, like the number of nuclear families, preventing associations in this scope.

## Conclusion

The results obtained in this study showed the high prevalence of violence involving adolescents in the school context, both as a victim and as an aggressor. The fact that this violence occurs in the classroom and colleagues witness and do not show protective actions to the victim is important aspects. The age of 12 to 14 years old and the male gender were significantly associated with the violence suffered, while the low education level of the mother was associated with the violence practiced. Also, the violence suffered or practiced showed no significant association with the use of alcohol, tobacco, and other drugs, despite the high prevalence of this behavior among adolescents. The use of other drugs was associated with the age group of 15 to 18 years old, and it was verified the sale of alcoholic beverages to children under 18 years old.

This worrying scenario leads to reflection on the effectiveness of public policy actions directed at confronting and preventing school violence among adolescents. In this sense, the present study brings significant additional knowledge to the current context of the phenomenon of violence in the school environment, which may be a base for future actions aimed at the development of healthy social skills, crucial for the development of adolescents. Also, looking at family members, educators and peers is necessary.
